# Secondary Household Transmission of 2009 Pandemic Influenza A (H1N1) Virus among an Urban and Rural Population in Kenya, 2009–2010

**DOI:** 10.1371/journal.pone.0038166

**Published:** 2012-06-11

**Authors:** Clara Y. Kim, Robert F. Breiman, Leonard Cosmas, Allan Audi, Barrack Aura, Godfrey Bigogo, Henry Njuguna, Emmaculate Lebo, Lilian Waiboci, M. Kariuki Njenga, Daniel R. Feikin, Mark A. Katz

**Affiliations:** 1 Epidemic Intelligence Service, Office of Workforce and Career Development, Centers for Disease Control and Prevention, Atlanta, Georgia, United States of America; 2 State Epidemiology Office, Ohio Department of Health, Columbus, Ohio, United States of America; 3 Global Disease Detection Division, Kenya Medical Research Institute/Centers for Disease Control and Prevention-Kenya, Kisumu and Nairobi, Kenya; 4 National Center for Immunization and Respiratory Diseases, Centers for Disease Control and Prevention, Atlanta, Georgia, United States of America; University of Hong Kong, Hong Kong

## Abstract

**Background:**

In Kenya, >1,200 laboratory-confirmed 2009 pandemic influenza A (H1N1) (pH1N1) cases occurred since June 2009. We used population-based infectious disease surveillance (PBIDS) data to assess household transmission of pH1N1 in urban Nairobi (Kibera) and rural Lwak.

**Methods:**

We defined a pH1N1 patient as laboratory-confirmed pH1N1 infection among PBIDS participants during August 1, 2009–February 5, 2010, in Kibera, or August 1, 2009–January 20, 2010, in Lwak, and a case household as a household with a laboratory-confirmed pH1N1 patient. Community interviewers visited PBIDS-participating households to inquire about illnesses among household members. We randomly selected 4 comparison households per case household matched by number of children aged <5. Comparison households had a household visit 10 days before or after the matched patient symptom onset date. We defined influenza-like illnesses (ILI) as self-reported cough or sore throat, and a self-reported fever ≤8 days after the pH1N1 patient's symptom onset in case households and ≤8 days before selected household visit in comparison households. We used the Cochran-Mantel-Haenszel test to compare proportions of ILIs among case and comparison households, and log binomial-model to compare that of Kibera and Lwak.

**Results:**

Among household contacts of patients with confirmed pH1N1 in Kibera, 4.6% had ILI compared with 8.2% in Lwak (risk ratio [RR], 0.5; 95% confidence interval [CI], 0.3–0.9). Household contacts of patients were more likely to have ILIs than comparison-household members in both Kibera (RR, 1.8; 95% CI, 1.1–2.8) and Lwak (RR, 2.6; 95% CI, 1.6–4.3). Overall, ILI was not associated with patient age. However, ILI rates among household contacts were higher among children aged <5 years than persons aged ≥5 years in Lwak, but not Kibera.

**Conclusions:**

Substantial pH1N1 household transmission occurred in urban and rural Kenya. Household transmission rates were higher in the rural area.

## Introduction

2009 pandemic influenza A (H1N1) (pH1N1) virus was responsible for at least 20,000 laboratory-confirmed deaths globally [1]. The first-recognized case of laboratory-confirmed pH1N1 in Kenya was identified on June 29, 2009, and by September 2009 a majority of influenza cases in the country were caused by pH1N1 [2].

Household transmission patterns of influenza infections vary by specific circulating strains; secondary attack rates of influenza among households range from 10% to 40% [3]–[7]. For pH1N1, studies conducted in the United States demonstrated secondary household attack rates of 9% and 11% for influenza-like illness (ILI) [8], [9], 13% for acute respiratory illness and 4% for laboratory-confirmed cases [10]. Other studies reported secondary household attack rates of 14.5% for ILI or laboratory-confirmed cases in Australia [11], and 8% for laboratory-confirmed cases in Hong Kong [12]. Because of their age and lack of prior exposure to years of circulating influenza viruses, children are more susceptible to infection with seasonal influenza viruses. Studies conducted in the United States and the United Kingdom demonstrated that children were more susceptible to pH1N1 compared with adults [8]–[10], [13], [14]. Children are also thought to be more infectious than adults because they tend to spend a lot of time in proximity to others [15], [16]. In contrast to seasonal influenza outbreaks, an evaluation of transmission during the first months after emergence of pH1N1 did not find age to be associated with infectiousness [9].

Transmission patterns of pH1N1 have been studied most closely in North America, Europe, and East Asia [8]–[10], [12]–[14], [17]–[20]. Knowledge is limited regarding transmission of pH1N1 in Africa, where comorbidities (e.g., HIV infection, malaria, and nutritional deficiencies) are more common than other locations. The effect of population density on pH1N1 transmission patterns has not been studied. We assessed and compared secondary attack rates of pH1N1 among households in urban and rural Kenya by using data from an ongoing rigorous, population-based infectious disease surveillance system, and we explored the role of age in household transmission.

## Methods

### Ethics Statement

The protocol was reviewed and approved by the Ethical Review Boards of the Kenya Medical Research Institute (KEMRI) (SSC#932) and the Institutional Review Board of the US Centers for Disease Control and Prevention (CDC) (IRB# 4566).

### Study Regions

The Centers for Disease Control and Prevention-Kenya (CDC-K) and KEMRI have been collaboratively conducting population-based infectious disease surveillance (PBIDS) for pneumonia, diarrhea, fever and jaundice since late 2005 in 2 regions in Kenya: Kibera, a large, informal urban settlement in Nairobi, and Lwak, a rural area in western Kenya. The study regions and surveillance methods have been described previously [21], [22]. Kibera has a population density of ∼70,000 persons/km^2^. Homes are single-level, built with mud, wood, and metal sheeting, chaotically scattered along unpaved roads and open sewers. A majority of men work as security guards, servants, casual laborers, or small business merchants within the city [21]. The altitude of Nairobi is approximately 1,600 meters, minimizing the likelihood for substantial transmission of malaria. Lwak has a population density of ∼325 persons/km^2^. Multi-family compounds are spread throughout the area, often with great distance between compounds. Residents are predominantly farmers and fishermen. Malaria is endemic. Approximately 16% of febrile patients in Kibera have malaria parasitemia (CDC-Kenya, unpublished data), whereas >50% of ILI cases in Lwak are associated with malaria-positive blood smears [23]. Temperatures are different in the two sites; Lwak is hotter and has more rainfall compared to Nairobi. The daily mean temperature in Nyanza, the province where Lwak is located, ranges from a high of 30.8°C in February to a low of 27.7°C in July. Annual rainfall is approximately 1,400 mm/year, peaking in April–May and November–December. In contrast, mean daily maximum temperature in Nairobi ranges from a high of 25.6°C in February and March to a low of 20.6°C in July. Annual rainfall in Nairobi averages approximately 1000 mm/year with peak rainfall in April–May and November–December [24].

### Household Morbidity Surveillance

Approximately 28,000 and 25,000 persons participated in PBIDS in Kibera and Lwak, respectively. Community interviewers visited participating households regularly to inquire about illnesses among household members. For the household morbidity surveillance, community interviewer visits were conducted biweekly in Kibera until September 2009, when visits were increased to weekly across half of the study site. Beginning February 1, 2010, community interviewer household visits were conducted weekly across the entire study site. In Lwak, household visits were conducted biweekly until they were increased to weekly on January 4, 2010. During household visits, community interviewers asked participants if they had experienced cough, fever, diarrhea, and other symptoms since the previous visit. If the resident reported currently or previously having any symptom since the previous visit, the community interviewer collected detailed information about symptom onset and duration, measures temperature, 1-minute respiratory rate, and, among children, evaluated lower chest wall indrawing and stridor.

### Clinic-Based Surveillance

Each surveillance site had 1 field clinic that provided free medical care to all study residents. In Kibera, residents could attend Tabitha Clinic, an outpatient facility owned by Carolina for Kibera (Chapel Hill, NC) and staffed and equipped by KEMRI-CDC. In Lwak, residents could attend St. Elizabeth Lwak Mission Hospital, which had inpatient and outpatient facilities. All participants lived within 1 and 5 kilometers from the clinics in Kibera and Lwak, respectively. Sick residents who visited the clinic were questioned regarding symptoms of present illness. Additionally, information about vital signs, physical exam, diagnosis, treatment, and outcome were collected along with specimens from patients meeting certain clinical criteria.

During the study period, nasopharyngeal and oropharyngeal swabs were collected at field clinics from patients with ILI or acute lower respiratory illness (ALRI). ILI was defined as an axillary temperature ≥38°C and cough or sore throat [25]. The ALRI case definition differed by patient age. ALRI for persons aged <5 years was defined, according to the World Health Organization's Integrated Management of Childhood Illness guidelines for severe pneumonia [26], as cough or difficulty breathing, and 1 of the following symptoms: inability to drink or breastfeed, convulsions, lower chest indrawing, loss of consciousness, lethargy, vomiting, stridor, or blood oxygen saturation <90%. In persons aged ≥5 years, ALRI was defined as an axillary temperature ≥38°C or blood oxygen saturation <90%, and either cough, difficulty breathing, or chest pain. All specimens were processed at the KEMRI-CDC International Emerging Infections Program Laboratory in Nairobi. Specimens were tested for influenza A and B by real-time reverse transcriptase-polymerase chain reaction (RT-PCR); those testing positive for influenza A were further subtyped by RT-PCR. The RT-PCR testing used the CDC pH1N1 testing protocol [27]. Specifics of the laboratory testing were described previously [28].

### Patient and Case Household

A pH1N1 case was defined as any laboratory-confirmed pH1N1 infection in an ILI or ALRI patient evaluated at the field clinic during August 1, 2009–February 5, 2010 in Kibera or August 1, 2009–January 20, 2010 in Lwak. The study periods were determined on the basis of available data at the time of analyses. Only patients who had a household visit ≤20 days after their symptom onset date were included in the study. Date of symptom onset was calculated by using data from the clinic questionnaire. The most common reason that patients did not have an interview in the 20 days after symptom onset was that the patient or household proxy was not at home during the community interviewer visit. In households with ≥1 laboratory-confirmed pH1N1 illness, the person with the earliest symptom onset date was considered the index patient. A case household was defined as a household with a laboratory-confirmed pH1N1 patient; a case-household person was a household contact of the pH1N1 patient.

### Comparison Household

To estimate the underlying illness rate in the community, we randomly selected from the PBIDS database 4 comparison households per case household, matched by the number of children aged <5 years. Comparison households did not have laboratory-confirmed pH1N1 cases, and had to have had a household interview ≤10 days before or after the matched patient symptom onset date.

### Secondary Illness

We calculated the number of ILI cases among case-household and comparison-household persons by using data from household visits. For case-household persons, we considered laboratory-confirmed pH1N1 infections or episodes of ILI that occurred ≤8 days after the index patient's symptom onset date as a secondary cases. The cutoff of 8 days was determined on the basis of average duration of pH1N1 shedding in Kibera as determined in a recent study [28]. For comparison household members, a case was defined as an episode of ILI that occurred ≤8 days before the household morbidity surveillance interview date.

### Statistical Analysis

We compared the sex distribution of the case and comparison households by using the Cochran-Mantel-Haenszel (CMH) test, adjusted for the matching factor. We compared the age and family size of the 2 groups by using the Wilcoxon signed-rank test for matched samples. We calculated the age-adjusted risk ratio (aRR) and corresponding 95% confidence interval (CI) of secondary ILIs in index households in Kibera and Lwak by using log-binomial regression [29]. We calculated the risk ratio (RR) and its corresponding 95% CI of secondary ILIs among case households and cases of ILI among comparison households. We assessed the role of age in pH1N1 transmission with respect to susceptibility (e.g., getting infected) and infectiousness (e.g., ability to infect others). For susceptibility, we compared the proportion of secondary ILIs among household members aged <5 years with those aged ≥5 years by calculating RRs and corresponding 95% CIs. To determine the age group that was most infectious, we categorized pH1N1 patients into 3 age groups (<5, 5–15, and ≥15 years) and compared the proportion of secondary ILIs associated with the index pH1N1 patient among each age group by using chi-square tests.

## Results

### Patients

**Figure 1:**
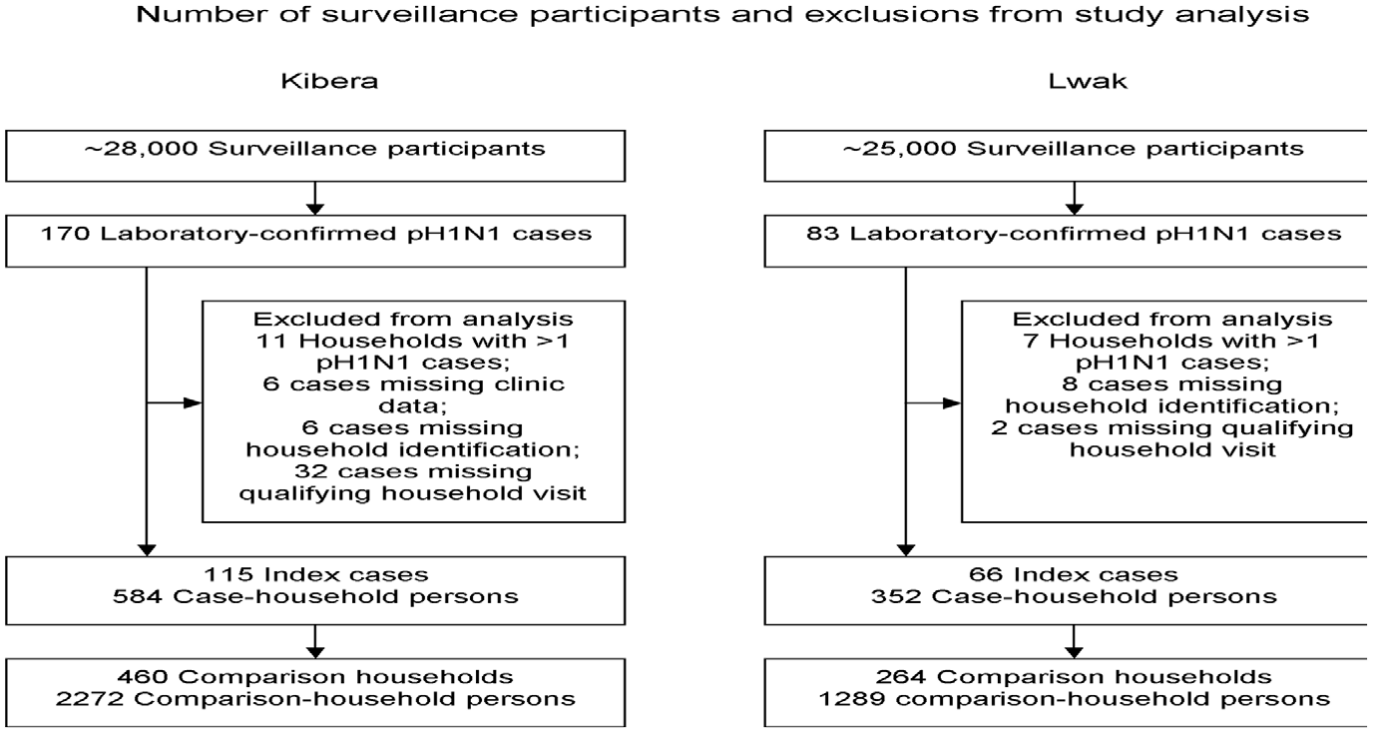
Number of surveillance participants and exclusions from study analysis – Kibera and Lwak, Kenya, 2009–2010.

We identified 170 laboratory-confirmed pH1N1 infections in Kibera and 83 in Lwak. As presented in Figure 1, among the 170 laboratory-confirmed Kibera cases, 55 cases were excluded from the study because of missing household identification information (6); missing clinic data (6); because no household interview occurred ≤20 days after symptom onset (32); or because they were not the first laboratory-confirmed case in the household (11). Among the 83 laboratory-confirmed Lwak cases, 17 were excluded because of missing household identification information (8); because no household interview occurred ≤20 days of symptom onset (2); or because they were not the initial laboratory-confirmed case in the household (7). Therefore, we included 115 (Kibera) and 66 (Lwak) patients, in our analysis.

**Figure 2:**
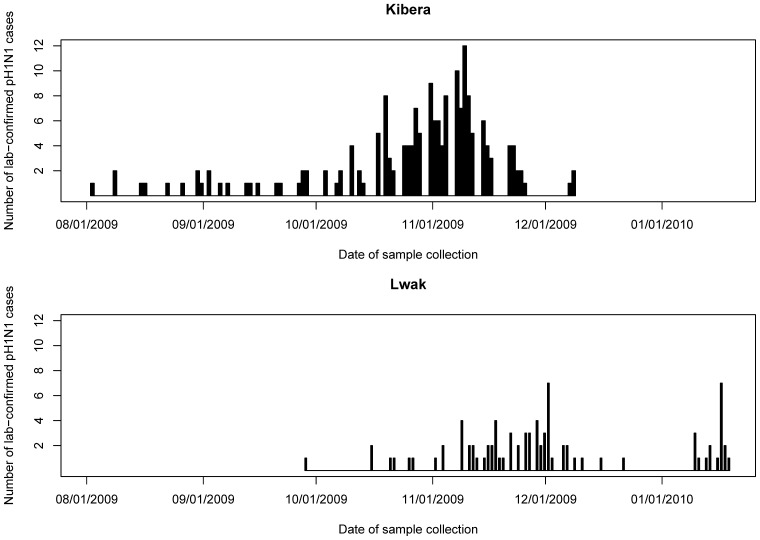
Number of laboratory-confirmed **pH1N1** cases by sample collection date – Kibera and Lwak, Kenya, 2009–2010. The x-axis indicates the sample collection date, and the y-axis indicates the number of lab-confirmed pH1N1 cases.

In Kibera, the median (range) age among patients was 7.1 (0.6–41.3) years, and 59 (51%) were female (Table 1). In Lwak, the median (range) age was 9.7 (0.2–55.9) years, and 35 (53%) were female (Table 2). The first laboratory-confirmed pH1N1 infections were confirmed on August 3 and September 29, 2009, in Kibera and Lwak, respectively (Figure 2). The number of laboratory-confirmed cases in Kibera was highest in early November and decreased thereafter. Lwak’s initial peak was during late November, approximately a week later than that in Kibera, and increased again at the end of December.

**Table 1:** Demographics of Laboratory-Confirmed pH1N1 Patients, and Case Household and Comparison Household Persons, and Family Size of Case Households and Comparison Households – Kibera, Kenya, 2009–2010.

	Patients	Case household persons	Comparison household persons	*P* values^a^
	n = 115	n = 584	n = 2272	
Sex				
Female	59 (51.3%)	311 (53.3%)	1175 (51.7%)	.62
Age (yrs)				
Mean (Std)^b^	10.2 (8.6)	19.7 (14.2)	18.4 (14.1)	.31
Median	7.1	16.6	15.8	
Min^c^, max^d^	0.6, 41.3	0.1, 67.5	0.04, 70.2	
		Index household	Comparison household	
		n = 115	n = 460	
Family size^e^				
Mean (Std)^b^		5.1 (2.4)	4.9 (2.5)	.55
Median		5.0	5.0	
Min^c^, max^d^		1, 15	1, 17	

a We calculated the *P*-values to compare case and comparison households by using Cochran-Mantel-Haenszel test for sex and age group, and Wilcoxon signed rank test for age and family size.

b Std  =  standard deviation.

c Min  =  minimum.

d Max  =  Maximum.

e Analyses of case households family size does not include the index cases.

**Table 2:** Demographics of Laboratory-Confirmed pH1N1 Patients, and Case Household and Comparison Household Persons, and Family Size of Case Households and Comparison Households – Lwak, Kenya, 2009–2010.

	Patients	Case household persons	Comparison household persons	*P* values^a^
	n = 66	n = 352	n = 1289	
Sex				
Female	35 (53.0%)	176 (50.0%)	674 (52.3%)	.78
Age (yrs)				
Mean (Std)^b^	11.7 (9.9)	20.2 (16.4)	22.1 (19.2)	.19
Median	9.7	15.5	16.6	
Min^c^, max^d^	0.2, 55.9	0.1, 87.5	0, 90.4	
		**Case household**	**Comparison household**	
		n = 66	n = 264	
Family size^e^				
Mean (Std)^b^		5.3(2.9)	4.9 (2.3)	.06
Median		4.5	5.0	
Min^c^, max^d^		1, 17	1, 12	

a We calculated the *P*-values to compare case and comparison households by using Cochran-Mantel-Haenszel test for sex and age group, and Wilcoxon signed rank test for age and family size.

b Std  =  standard deviation.

c Min  =  minimum.

d Max  =  Maximum.

e Analyses of case households family size does not include the index cases.

### Case Households and Comparison Households

In Kibera, 311 (53%) and 1175 (52%) of the case household and comparison household members were female, respectively. The median (range) age was 16.6 (0.1–67.5) and 15.8 (0.04–70.2) years among case and comparison households, respectively (Table 1). In Lwak, 176 (50%) and 674 (52%) of the case and comparison household members were female, respectively. The median (range) age was 15.5 (0.1–87.5) and 16.6 (0–90.4) years, among case and comparison households, respectively (Table 2).

### Proportion of Influenza-Like Illnesses Among Case Households and Comparison Households

In Lwak, 8.2% of the case household members had secondary ILI, whereas 3.3% of the comparison household members had ILI (RR, 2.6; 95% CI, 1.6–4.3). In Kibera, 4.6% of the case household members had secondary ILI, whereas 2.7% of the comparison household members had ILI (RR, 1.8; 95% CI, 1.1–2.8) (Table 3). The proportion of secondary ILI cases among case households was significantly lower in Kibera compared with Lwak, (aRR, 0.5; 95% CI, 0.3–0.9). The mean (standard deviation) serial interval was 4.0 (1.6) and 3.2 (3.0) in Kibera and Lwak, respectively.

**Table 3:** Number (%) of Secondary Influenza-Like Illness Among Case Households and Influenza-Like Illness Among Comparison Households – Kibera and Lwak, Kenya, 2009–2010.

Location	Case-household persons	Comparison-household persons	Relative risk (95% CI^a^)
			
Kibera	n = 584	n = 2,272	
	27 (4.6%)	62 (2.7%)	1.8 (1.1–2.8)
Lwak	n = 352	n = 1289	
	29 (8.2%)	43 (3.3%)	2.6 (1.6–4.3)

a CI  =  confidence interval.

### Secondary Influenza-Like Illness by Household Member Age

The proportion of secondary cases of ILI among case households was greater among children aged <5 years than among persons aged ≥5 years in both sites (Table 4). In Lwak, the difference was statistically significant; children aged <5 years were 3.8-fold more likely to have had a secondary case of ILI than persons aged ≥5 years (22.5% versus 5.9%; RR 95% CI, 1.9–7.5). In Kibera, children aged <5 years were 1.4-fold more likely to have had a secondary case of ILI compared with persons aged ≥5 years, but the difference was not statistically significant (6.1% versus 4.3%; RR 95% CI, 0.6–3.4).

**Table 4:** Percentage (number) of Secondary Influenza-Like Illness Among Household Contacts of Confirmed pH1N1 patients, by Household Contact Age and by Patient Age – Kibera and Lwak, Kenya, 2009–2010.

	Location	
Age	Kibera	Lwak
Household contact		
<5	6.1% (6/98)	4.3% (21/486)
≥5	22.5% (11/49)	5.9% (18/303)
Relative Risk (95%CI a)	1.4 (0.6–3.4)	3.8 (1.9–7.5)
Patient		
<5	5.0% (11/219)	3.2% (2/62)
5–15	3.3% (8/245)	11.2% (23/206)
≥15	6.7% (8/120)	4.8% (4/84)
P-value^b^	.33	.06

a CI = confidence interval.

b We calculated the p-values using chi-square tests.

### Secondary Influenza-Like Illness by Index Patient Age

In both sites, the proportion of secondary cases of ILI differed according to the index pH1N1 patient age group, but the difference was not statistically significant in either site (*P*  = .33 in Kibera, and *P*  = .06 in Lwak) (Table 4). In Kibera, the proportion of secondary ILI was highest when the index patient was aged ≥15 years (6.7%, 8/120), second-highest when the index patient was aged <5 years (5.0%, 11/219), and lowest when the index patient was aged 5–15 years (3.3%, 8/245). In Lwak, the proportion of secondary ILI was highest when the index patient was aged 5–15 years (11.2%, 23/206), second-highest when the index patient was aged ≥15 years (4.8%, 4/84), and lowest when the index patient was aged <5 years (3.2%, 2/62).

## Discussion

To our knowledge this is the first prospective study to evaluate pH1N1 transmission dynamics in Africa. We found that households with laboratory-confirmed pH1N1 cases had a substantially higher proportion of ILI compared with households without a laboratory-confirmed case in both urban and rural Kenya.

The absolute pH1N1 secondary household attack rates (4.6% in Kibera and 8.2% in Lwak) were comparable to findings from pH1N1 transmission studies in the United States and Hong Kong [9], [10], [18]. A study conducted in Hong Kong determined a secondary illness rate for pH1N1 of 5.9% when the patients were secondary school students and secondary cases were defined as acute respiratory illness and laboratory-confirmed pH1N1 [18]. A study in the United States (Texas) found secondary attack rates of 4% for laboratory-confirmed pH1N1 infection and 9% for ILI [10]. In a New York City high school, 11.3% of households with at least 1 laboratory-confirmed pH1N1 case had secondary ILI cases [9]. In contrast, a study in Quebec City, Canada, identified a much higher secondary household attack rate of 45% for laboratory-confirmed pH1N1, and 81% of households with an pH1N1 case had ≥1 secondary case [19]. Two additional studies reported higher pH1N1 secondary household attack rates than our study; a study in Edmonton, Canada reported that in 28.7% of households with a laboratory-confirmed pH1N1 patient, at least one household member developed secondary ILI, and in 22.9% of these same households, at least 2 household members developed secondary ILI [20]; and a study in Australia reported a secondary ILI rate of 33% among household contacts of laboratory-confirmed pH1N1 patients [30].

The proportion of secondary ILI cases among case households was greater in rural Lwak than in urban Kibera. This might be explained in part by lack of specificity in identifying secondary ILI cases. Malaria infections in Lwak might have contributed to the higher rate of secondary ILIs in Lwak, where malaria is endemic, compared with Kibera, where malaria is nonendemic. A recent study reported that 65.7% of ILI patients in Lwak had blood smear-positive malaria [23]. Household members with malaria who had fever and a cough or sore throat would have met our syndromic ILI case definition. In addition, other viral or bacterial causes of ILI may have varied in the two sites and could have contributed to the difference in the proportions of secondary ILIs. Antiviral medications were only used by two laboratory-confirmed cases in Kibera, and were not used in Lwak [28]. Therefore, antiviral medications were unlikely a factor in virus transmission in the two sites.

Transmission rates did not significantly differ according to the age of the patient. These findings are similar to those from a study of pH1N1 transmission in the United States conducted in the first months after pH1N1 emerged, which found that infectiousness was not associated with patient age [8]. In contrast, a household-transmission study of seasonal influenza demonstrated that household members exposed to preschool or school-aged patients had an increased risk for secondary illness compared with those exposed to adult patients [15]. These findings might have relevance for pH1N1 immunization strategies. Although a strategy to use limited resources to minimize virus transmission by targeting children might be useful for seasonal influenza based on past studies, our findings suggest that in Kenya, a pH1N1 immunization campaign initially focused on children may not disproportionately prevent pH1N1 transmission.

In the U.S. study, children were twice as susceptible to infection with pH1N1 virus, compared with adults [8]. In Kenya, the magnitude of susceptibility among children differed by population. Children aged <5 years who were contacts of laboratory-confirmed pH1N1 patients were approximately 4 times more likely to acquire secondary illness compared with older contacts in the rural site; however, susceptibility was not associated with age of contacts in the urban site. Again, this might be related to the high prevalence of malaria in Lwak. In endemic settings, malaria causes more symptomatic infections among children than among adults [31]. Therefore, in Lwak, malaria infections among children might have contributed to the number of secondary ILI cases, and therefore could have been a confounder in the susceptibility analysis.

Unlike published pH1N1 household transmission studies, our study used data from an ongoing population-based surveillance system in which study participants were recruited before the pandemic occurred. Therefore, the disease status of individuals among the households did not affect their decision to participate in this study. However, our study had certain limitations. We used self-reported ILI rather than laboratory-confirmed influenza among secondary cases to estimate transmission, and consequently might have classified illnesses caused by something other than pH1N1 as secondary pH1N1 cases, especially in malaria-endemic Lwak. Also, we might have missed secondary cases of pH1N1 that did not meet the ILI definition. However, the proportion of missed pH1N1 cases should have been similar among case households and comparison households. We may not have captured all secondary pH1N1 infections; 28% and 26% of the laboratory-confirmed cases in Kibera and Lwak, respectively, would not have met our secondary ILI definition if data from household visits were used in the case definition for patients. Our study could not distinguish secondary ILIs attributable to direct transmission from a confirmed pH1N1 patient and illnesses acquired outside the household or from a household member other than the confirmed patient. In Kibera, inter-household interactions, given the population density, likely occur much more frequently than in Lwak, and therefore in Kibera influenza transmission may occur more commonly in the community rather than in the household. Differences in social interaction and mixing have been shown to have an impact on influenza transmission in previous studies [32], and these differences may have also played a role in the transmission of pandemic influenza in Kibera and Lwak. However, we did not collect data on social interaction and mixing and therefore we were unable to evaluate these factors in the context of this study. In addition, households that had a member ill enough to seek medical treatment might have been more likely to recall illnesses among other household members than households that did not seek medical treatment. Household interviews were conducted biweekly in half of the study site in Kibera, and all of Lwak for the majority of the study period. A previous description of recall bias from these two surveillance sites found that participants’ reporting of symptoms diminished significantly after 4 symptom-free days [21]. Because of the long interval between household interviews, participants might have had difficulty recalling their symptoms. However, for our study, we included ILI cases that occurred up to 8 days before the interview in comparison households and ILI that occurred up to 8 days after the patient's symptom onset in case households, regardless of when the interview occurred. Therefore, when considering the lag between symptom onset and interview, case household members might have had more difficulty recalling illness than comparison household members. Such a limitation would have biased toward the null, potentially minimizing our secondary transmission rate calculations.

We found that pH1N1 household transmission occurred in Kenya at similar rates to what has been reported in other studies in more developed countries. However, in Kenya, secondary transmission patterns differed in urban and rural environments. Children were not significantly more likely to transmit pH1N1 than older persons, a characteristic of pH1N1 transmission patterns that differed from seasonal influenza.
