# Correction: Secondary Household Transmission of 2009 Pandemic Influenza A (H1N1) Virus among an Urban and Rural Population in Kenya, 2009–2010

**DOI:** 10.1371/journal.pone.0091443

**Published:** 2014-03-26

**Authors:** The PLOS ONE Staff

In Table 4, the second row in the column titled "Lwak" should be 22.5% (11/49), instead of 4.3% (21/486). Additionally, the third row of the column titled "Kibera" should be 4.3% (21/486), instead of 22.5% (11/49). Please see the correct Table 4 here:

**Table 4:** Percentage (number) of Secondary Influenza-Like Illness Among Household Contacts of Confirmed pH1N1 patients, by Household Contact Age and by Patient Age - Kibera and Lwak, Kenya, 2009-2010.

Age	Location	
	Kibera	Lwak
Household contact		
<5	6.1% (6/98)	22.5% (11/49)
≥5	4.3% (21/486)	5.9% (18/303)
Relative Risk (95% CI^ a^)	1.4 (0.6–3.4)	3.8 (1.9–7.5)
Patient		
<5	5.0% (11/219)	3.2% (2/62)
5–15	3.3% (8/245)	11.2% (23/206)
≥15	6.7% (8/120)	4.8% (4/84)
P-value^b^	.33	.06

a CI = confidence interval.

b We calculated the p-values using chi-square tests.

## References

[1] Kim CY, Breiman RF, Cosmas L, Audi A, Aura B, et al (2012) Secondary Household Transmission of 2009 Pandemic Influenza A (H1N1) Virus among an Urban and Rural Population in Kenya, 2009–2010. PLoS ONE 7(6): e38166 doi:10.1371/journal.pone.0038166 22701610 10.1371/journal.pone.0038166 PMC3372521

